# Combination Therapy of Mithramycin A and Immune Checkpoint Inhibitor for the Treatment of Colorectal Cancer in an Orthotopic Murine Model

**DOI:** 10.3389/fimmu.2021.706133

**Published:** 2021-07-26

**Authors:** Rinku Dutta, Roukiah Khalil, Karthick Mayilsamy, Ryan Green, Mark Howell, Srinivas Bharadwaj, Shyam S. Mohapatra, Subhra Mohapatra

**Affiliations:** ^1^ James A. Haley Veterans’ Hospital, Tampa, FL, United States; ^2^ Department of Molecular Medicine, Morsani College of Medicine, University of South Florida, Tampa, FL, United States; ^3^ Center for Research and Education in Nano-Bioengineering, Morsani College of Medicine, University of South Florida, Tampa, FL, United States; ^4^ Department of Internal Medicine, Morsani College of Medicine, University of South Florida, Tampa, FL, United States

**Keywords:** PD-L1, orthotopic tumor, colorectal cancer, Mithramycin-A, combination therapy

## Abstract

The axis of Programmed cell death-1 receptor (PD-1) with its ligand (PD-L1) plays a critical role in colorectal cancer (CRC) in escaping immune surveillance, and blocking this axis has been found to be effective in a subset of patients. Although blocking PD-L1 has been shown to be effective in 5–10% of patients, the majority of the cohorts show resistance to this checkpoint blockade (CB) therapy. Multiple factors assist in the growth of resistance to CB, among which T cell exhaustion and immunosuppressive effects of immune cells in the tumor microenvironment (TME) play a critical role along with other tumor intrinsic factors. We have previously shown the polyketide antibiotic, Mithramycin-A (Mit-A), an effective agent in killing cancer stem cells (CSCs) *in vitro* and *in vivo* in a subcutaneous murine model. Since TME plays a pivotal role in CB therapy, we tested the immunomodulatory efficacy of Mit-A with anti-PD-L1 mAb (αPD-L1) combination therapy in an immunocompetent MC38 syngeneic orthotopic CRC mouse model. Tumors and spleens were analyzed by flow cytometry for the distinct immune cell populations affected by the treatment, in addition to RT-PCR for tumor samples. We demonstrated the combination treatment decreases tumor growth, thus increasing the effectiveness of the CB. Mit-A in the presence of αPD-L1 significantly increased CD8^+^ T cell infiltration and decreased immunosuppressive granulocytic myeloid-derived suppressor cells and anti-inflammatory macrophages in the TME. Our results revealed Mit-A in combination with αPD-L1 has the potential for augmented CB therapy by turning an immunologically “cold” into “hot” TME in CRC.

## Introduction

Colorectal cancer (CRC) is the third most common cancer in men, the second most common in women, and the second most common cause of cancer-related deaths in the USA (1). With an estimated 5% lifetime risk, CRC is one of the malignant cancers whose 5-year survival rate is poor when patients are diagnosed at a late stage (2, 3). Microsatellite instability (MSI) plays a pivotal role in CRC stages and arises due to deficiencies in the DNA mismatch repair system, causing insertion, deletion, or misincorporation of nucleotides in the DNA (4). Recent advances in checkpoint blockade (CB) therapy for microsatellite instability (MSI) positive CRC patients have shown dramatic response for patients with high MSI (MSI-H) (5). Current FDA-approved combination immunotherapy drugs used for metastatic CRC are ipilimumab (Yervoy) and nivolumab (Opdivo) for patients with MSI-H (6, 7). However, single-agent checkpoint inhibitors do not show response in CRC patients with microsatellite stable (MSS) carcinomas, which comprise the majority of the aggressive CRCs with poor outcomes (5, 8).

Programmed death-1 (PD-1) is a checkpoint molecule that is highly expressed on tumor-infiltrating T cells. PD-1 ligand (PD-L1) is variably expressed on tumor cells and tumor-infiltrating antigen-presenting cells and is considered a negative prognostic marker (9). Engagement of PD-1 with PD-L1 suppresses T cell response and inhibits anti-tumor immunity (10). Hence, attempts are made to use checkpoint blocking antibodies against PD-1/PD-L1 as promising immunotherapy in CRC treatment. Unfortunately, patients with MSS showed 0% response to anti-PD-1 CB therapy (5, 11).

PD-L1 is a transmembrane protein belonging to the B7 family of the Ig superfamily and are expressed on lymphocytes (B and T), NK cells, dendritic cells, as well as IFN-γ stimulated monocytes, epithelial cells, and endothelial cells (12). Engagement of PD-L1 with PD-1 leads to inhibition of both T cell proliferation and cytokine production (8, 13). Thus, PD-L1 is thought to play an essential role in tumor immune evasion. Further, elevated PD-L1 expression has been found in some tumors resulting in increased resistance of tumor cells to CD8^+^ T cell-mediated lysis (9). Thus, inhibiting PD-L1 with its antibody forms one of the bases of CB therapies. However, as the application of CB monotherapy has failed in MSS patients, combination approaches with chemo-drugs hold potential as a sensitizer to anti-tumor immune cells along with immune modulation (5). Also, combination therapies can lead to increased immune T-cell infiltration, which is generally found in MSI patients responsive to the treatment (4). As a result, combination chemotherapy strategies are evolving with CB for the treatment of metastatic CRC (14, 15).

The tumor microenvironment (TME) is infiltrated with immunosuppressive myeloid-derived suppressor cells (MDSCs) that constitute part of the myeloid regulatory network (16). In CRC, these cell types along with tumor-associated macrophages (TAMs) play a pivotal role in tumor immune evasion to cue the immune surveillance to bypass recognition of the tumor as foreign (16, 17). Therefore, they are often recruited to the TME, expanding and suppressing anti-tumor immunity (18).

Mithramycin A (Mit-A) is a polyketide antibiotic which is proven to bind to the minor groove of DNA and thus it inhibits the binding of the transcription factor SP1 with the DNA (19). Therefore, Mit-A potently inhibits the transcription factor, SP1, which is involved in chemoresistant cancers (20, 21). Moreover, it has been found to sensitize tumor cells to TRAIL-mediated apoptosis *via* XIAP-gene promoter downregulation *via* its SP1 sites (22). Recently, we have demonstrated Mit-A can specifically target cancer stem cells (CSCs) by inhibiting CSC proliferation when tested in mouse and human colon cancer tumor organoid (tumoroid) cultures (both *in vitro* and *ex vivo*) and *in vivo* (23).

We reasoned, that combining Mit-A with CB could increase the latter’s effectiveness in the complex milieu of TME. Since immunosuppressive cells such as MDSCs and TAMs contribute to reduced T cell infiltration and activation (24), we reasoned that this combination might target the PD-L1 on the tumor cells and the MDSC and TAM and thus promote anti-tumor immune activation. Since the PDL1 promoter region has been found to serve as a binding site for SP1 in gastric cancer and rs10815225 polymorphism is related to the overexpression of PD-L1 (25), we reasoned Mit-A (an SP1 inhibitor) could influence the PD-L1 expression in TME. In this context, we were interested in studying the effects of Mit-A treatment on the immune cells such as for MDSC and macrophage-mediated immunosuppression in the TME. We hypothesized that treatment of tumor cells with Mit-A would lead to sensitization to αPD-L1 therapy, thus increasing the efficacy of the PD-L1 CB. To test our hypothesis, we used an MC38 (p-53 mutant, K-RAS wild-type, MSI-H) orthotopic tumor-bearing mouse model and treated it with Mit-A combined with αPD-L1 mAb. We demonstrated treatment with Mit-A significantly increases the latter’s effectiveness by upregulating the PD-L1 of the granulocytic MDSCs and tumor cells, thus making them more susceptible to inhibition by anti-PD-L1 therapy. The inhibition of immunosuppressive cells leads to an increase of TME infiltration by anti-tumor T-cells. Based on these findings, we suggest that Mit-A can increase the efficacy of CB combination therapy.

## Materials And Methods

### Antibodies and Reagents

All reagents and antibodies are listed in **Tables 2A, B** ; **Supplementary Figure 4**. Gibco Dulbecco’s Modified Eagle Medium (DMEM), L-glutamine, Fetal bovine serum (FBS) were purchased from Thermo Fischer Scientific. Mycoplasma kit was purchased from Lonza.

### Cell Culture and Drug Treatments

MC38 cells (colon carcinoma epithelial cells derived from C57BL/6 mice; wt-KRAS, MSI-H, and p-53 mutant) were provided by Dr. Shari Pilon-Thomas (Moffitt Cancer Center) and were cultured in DMEM medium containing 2 mM L-glutamine, 0.1 mM nonessential amino acids, 1 mM sodium pyruvate, 100 U/ml penicillin, 100 mg/ml streptomycin, and 10% FBS. CT26 cells were maintained in complete RPMI media (100 U/ml penicillin, 100 mg/ml streptomycin, and 10% FBS). HT29 and HCT116 were maintained in McCoy’s complete media as per ATCC. All cells were maintained in an atmosphere containing 5% CO_2_ and at 37°C. Besides, cells were routinely checked for mycoplasma contamination. MC38-Luc stable cells were created in-house following the standard transfection and G418 selection protocol. These cells were derived from MC38 cells as detailed earlier. Briefly, MC38 cells plated in 24-well cell culture-treated plates, grown to 70–80% confluency were transfected with the luciferase gene (Addgene) using Lipofectamine^®^ 3000 (Invitrogen). Post 48 h of transfection, the cells were treated with selection antibiotic (G418) (Geneticin, Gibco, Invitrogen) (concentration—400 µg/ml obtained by antibiotic kill curve). Positive and negative control was maintained. Transfected cells were transferred to a 60 mm tissue culture plate. The cells were then plated in 96 well plates at 1 cell/well to form colonies from an individual cell. A suspension of 10 cells/ml was obtained by limiting dilution and forming colonies for 1–2 weeks. Single-cell colonies checked for luciferase activity. Next the clones were expanded to 6-well plates and then to tissue culture flasks. MC38-Luc clones were maintained in complete media with 400 µg/ml of G418 during cell culture.

For PD-L1 expressions analyses upon Mit-A treatment study, 1 × 10^5^ and 0.75 × 10^5^ HCT116 cells and 2 × 10^5^ and 1.5 × 10^5^ HT29 cells were grown as monolayer and tumoroids respectively. For monolayer, Mit-A was treated the next day (10 nM for HCT116 and 50 nM for HT29) and for tumoroid on Day 4 (25 nM for HCT116 and 100 nM for HT29). PD-L1 expressions were analyzed post 48 h of treatment with human PD-L1 and isotype control antibodies by Flow cytometry.

### Tumoroid Culture

Polymeric nanofiber scaffold was prepared, sterilized in ethanol and used for tumoroid culture as previously described (22). Tumoroids were cultured in a humidified incubator at 37°C in a 5% CO_2_ atmosphere. 3D Tumoroid formation was assessed using fluorescent microscopy (Olympus BX51) after nuclear staining with Nuc Blue dye (Thermo Scientific).

### Cell Viability Assay

Cell growth was quantified using the CellTiter-Glo^®^ Luminescent Cell Viability (Promega, G7572) assay. For MC38 monolayer culture, 4,000 cells were plated in a 96-well plate and treated the next day with Mit-A as indicated (n = 3). For scaffold culture, 3,000 cells from tumor biopsies were plated in a 96-well plate in 50 µl volume to stabilize cells on the scaffold (n = 3). The next day, 150 µl of fresh media was added. On day 4 of plating, cells were treated with Mit-A and αPD-L1 antibodies. According to the manufacturer's protocol for biopsy monolayers, 48 and 72 h after treatment, Cell Titer Glo reagent was added with media (1:1 ratio). The luminescence signal was read by an illuminometer (Synergy H4 hybrid reader; BioTek) in an opaque plate. For biopsy scaffolds, on Day 6, a CellTiter-Glo assay was performed.

### Annexin V

Annexin V assay of a monolayer of MC38 cell line and biopsies from orthotopic tumor-bearing C57BL/6 mice was performed to measure the early and late apoptosis upon treatment with Mit-A w/o αPD-L1 antibody. As discussed earlier, biopsy tumors were dissociated into a single cell suspension and plated in 6-well plates at a cell density of 1.5 × 10^5^/well in 2 ml of complete media. MC38 cell line was also plated similarly with the same cell density in complete media. The next day, diluted stock (DMSO) of Mit-A (300 and 600 nM) w/o αPD-L1 antibody (20 µg/ml) in complete media was added to the wells for both the monolayers (cell line and biopsies) and Annexin V (APC) assay was performed in 1× binding buffer with DAPI post 72 h of treatment. Similarly, for scaffolds also the same protocol was followed post 72 h of treatment.

### Co-Culture Experiments: Tumoroid-T Cell Coculture

Orthotopic MC38 biopsies were collected, dissociated with Miltenyi tumor dissociation kit and collected as a single cell suspension. Approximately 1.2 × 10^5^ cells were plated on pre-sterilized scaffolds in 1 ml media per well in non-treated 6-well plates as per our in-house protocol (23). The next day (Day 1), 3 ml of media was added. On Day 4, CD8^+^T cells isolated from the spleen of naïve C57BL/6 female mice using CD8a^+^ isolation kit were CFSE stained following manufacturer’s protocol and activated with CD3/CD28 microbeads and added to the tumoroids in the ratio 1:1 and 1:6 in the presence of 30 U/ml of IL-2. Mit-A dissolved in DMSO was added to the scaffolds (600 and 800 nM) post T cell addition on the same day. Cells were collected post 72 h addition of Mit-A and stained with CD45, CD3, CD8 flow antibodies, and data collected in BD LSRII. Data analyzed by FlowJo software (version 10.). DAPI was used for live/dead staining. For positive and negative controls, activated and non-activated CFSE-stained CD8^+^T cells were plated separately in 6 well plates and treated with IL-2 similar to the scaffold cultures and analyzed post 72 h along with the Mit-A treated samples.

### MDSC-T Cell Co-Culture

Spleen collected from C57BL/6 orthotopic tumor-bearing mouse was dissociated, lysed with ACK (Ammonium Chloride-Potassium) lysis buffer, and collected into a single-cell suspension. Next, CD11b^+^ cells were collected by positive selection using CD11b^+^ microbeads and LS column in a MidiMACs separator, stained with CD11b^+^ antibodies and sorted for Ly6G^+^ and Ly6G^-^ cells using Ly6G-antibody and DAPI for live/dead staining in the FACS Melody cell sorter. Spleen from naïve C57BL/6 mice was made. Next, the CD8^+^ T cells were collected and stained with CFSE and activated using CD3/CD28 activation beads as mentioned earlier. Finally, the sorted granulocytes and monocytes were co-cultured in 96-well round-bottomed plates with the activated CD8^+^ T (in the presence of IL-2 (30 U/ml)) cells in the ratio 1:3 (MDSC : CD8^+^T cells) for the CD8^+^T cell proliferation activity studies for immunosuppressive effects of MDSCs on CD8^+^T cell activation/proliferation (26). On the same day, Mit-A (600 nM) w/o αPD-L1 mAb (20 µg/ml) was treated to the co-culture and cells collected post 72 h of treatment were analyzed by flow cytometry. For positive and negative controls, activated and non-activated CFSE-stained CD8^+^T cells were plated separately in 96-well round-bottomed plates and treated with IL-2 similar to the co-cultures and analyzed post 72 h with the Mit-A treated samples.

### *In Vivo* Experiments

All experiments were performed in accordance with the IACUC of the University of South Florida. *Orthotopic tumor model:* Wild-type C57BL/6 mice (female, 6–8 weeks old; from Jackson Laboratory) mice were anesthetized with isoflurane inhalation and the cecum was exposed *via* a lower abdominal incision. Approximately 2 × 10^5^ MC38-Luc cells suspended in 50 µl of PBS were injected subserosally using a 30-G BD insulin syringe under the microscope (Day 0). Mice were monitored regularly and surgical clips were removed on Day 7. Images captured every week post 1 week of tumor inoculation by *in vivo* imaging system (IVIS) (Xenogen; Perkin Elmer (Caliper Life Sciences) following intraperitoneal administration of D-Luciferin. The treatment regimen includes an equivalent amount of DMSO in PBS for vehicle control, 150 µg/mouse for IgG (isotype) and αPD-L1 mAb, 1 mg/kg of Mit-A with or without αPD-L1 in PBS. Initial and final mice body weights were taken.

### Flow Cytometry

Spleens and tumors from *in vivo* experiments were harvested under sterile conditions. Tumors were sliced into 2–4 mm^3^ pieces post collection and proceeded for enzymatic digestion using the Miltenyi tumor dissociation kit. Tumors were dissociated into single-cell suspensions, RBCs (Red Blood Cells) were removed using an ACK lysing buffer. Spleens were made into single-cell suspension in PBS followed by lyses with ACK lysing buffer. Approximately 1 × 10^6^ tumor cells and splenocytes were incubated with Zombie in PBS for 20 min in the dark at RT (room temperature), followed by washing at 300*g* for 3 min and subsequently washed with FACS buffer (PBS with 10% heat-inactivated FBS and 2mM EDTA) and stained with relevant antibodies (Abs) for 30 min on ice in FACS buffer followed by washing. For intracellular staining of Foxp3, cells were labeled with all other Abs first except Foxp3, fixed (with 1× Mouse Foxp3 Fixation Buffer), permeabilized (with 1× Mouse Foxp3 Permeabilization Buffer using manufacturer’s protocol). Next washed with FACS buffer and stained with Foxp3 Ab for 20 min at RT, followed by washing, re-suspending in FACS buffer and analyzing immediately using an LSRII flow cytometer (BD Biosciences) and data analyzed using FlowJo software (version 10).

### Immunohistochemistry

Approximately 10-micron cryosectioned tumor samples were used for immunostaining. Sections were baked and boiled in antigen unmasking solution (Vector Laboratories Inc., Burlingame, CA; 1–100) for 45 min at 90°C. Post heat antigen retrieval, sections were treated with 3% hydrogen peroxide in water for 20 min, then the sections were blocked and permeabilized with 10% serum, 0.2% Triton X-100 in PBS for 1 h at RT. Following this, the sections were incubated with primary antibody solution (5% host serum, 0.1% Triton X-100 in PBS) at 4°C overnight. Following washing, sections were then sequentially incubated with biotinylated secondary antibody for 2 h at RT, avidin–biotin-peroxidase (ABC, 1:100 Vector Laboratories, Inc, Burlingame, CA) for 1 h at RT and DAB substrate solution (Vector Lab. Inc.) for 5 min. Finally, sections were washed, dried and cover-slipped with DPX mounting medium. All images including bright field ones were taken using a Keyence microscope (BZ-X710 Fluorescence microscope).

### q-RT-PCR

Snap frozen tumor samples were homogenized in a lysis buffer and the total RNA was isolated using RNeasy Plus Mini kit (27) (Qiagen; Cat. no. 74134) followed by removing the residual DNA by treating with DNAse I. Using Maxima Enzyme and 5× Reaction Mix (Thermo Scientific), cDNA was prepared from 1 ug of RNA. With the cDNA, qRT-PCR was performed in CFX384 Touch Real-Time PCR Detection System (Bio-Rad). Reactions run in triplicates (n = 3) with cycles 95°C for 3 min, followed by 45 cycles of 95°C for 10 s, 60°C for 1 min and 72°C for 15 s with the reaction mixture containing 1 ul of 5× all-in-one SYBR-master mix, 2.5 ul of RNAse free water and 0.5 µl of primers (**Table 1**; **Supplementary Figure 3**) and 1 µl of cDNA. β-actin was run as an internal control for all the genes. Finally, ΔΔ^−Ct^ values were calculated to measure each gene expression change.

### Statistical Analysis

Data analyzed in Graphpad Prism (version 8). All quantitative data were analyzed through mean ± S.E.M (standard error of the mean) by Student’s t-test and One-way ANOVA (Fischer’s LSD test) as stated for each experiment.

## Results

### Mit-A Causes Increased Early Apoptosis in Combination With CB by Sensitizing *Ex Vivo* Tumor Biopsy Cultures to CB *via* Enhancing PD-L1 Expression

**Figure 1:**
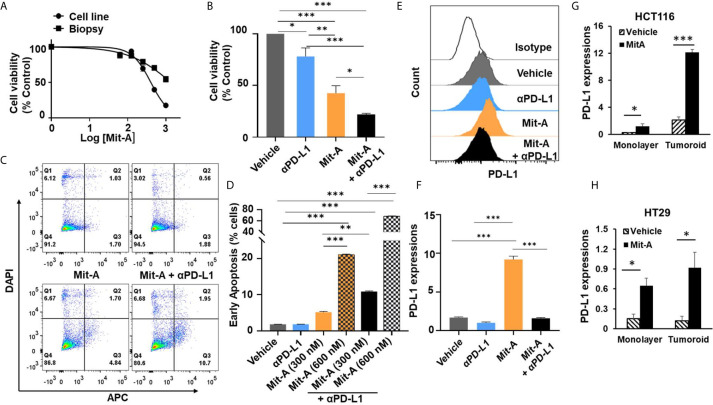
Mit-A combined with CB causes increased early apoptosis by sensitizing the orthotopic tumor *via* increased PD-L1 expression. **(A)** Cell viability assay for monolayer cultures of MC38 cell line or biopsies from orthotopic tumors by CellTiter-Glo (Promega) post 48 h of Mit-A treatment. **(B)** Cell viability of MC38 tumoroids of orthotopic biopsies grown on scaffold. On Day 4 tumoroids were treated with Mit-A (250 nM) with or without αPD-L1 mAb (10 µg/ml) and Cell Titer Glo assay was performed 48 h post treatment. **(C**, **D)** Annexin V-binding assay of monolayer culture of MC38 tumor biopsies treated with vehicle, Mit-A with or without anti-PD-L1 (αPD-L1) mAb (20 µg/ml) (post 72 h of treatment) where **(C)** depicts the representative images of Annexin-V binding assay; **(D)** represents the percentage of cells from the Annexin V-binding assay undergoing early apoptosis (Q3). **(E, F)** Mit-A treatment sensitizes the MC38 biopsy leading to the increased expression of PD-L1 which was reversed upon addition of the αPD-L1 mAb treatment where **(E)** depicts the histogram from the flow analysis and **(F)** represents the bar graph plot of PD-L1 expression of MC38 biopsy monolayer cells treated with Mit-A (300 nM) and αPD-L1 mAb (20 µg/ml). **(G, H)** represent the PD-L1 expressions for HCT116 and HT29 cells grown as monolayer and tumoroids upon treatment with Mit-A (10 and 50 nM for HCT 116 and HT29 monolayer) and (25 and 100 nM for HCT116 and HT29 tumoroids), respectively. IC50 values were analyzed by non-linear regression analysis in Graphpad prism (version 8). Data analyzed as mean ± SEM by One-way ANOVA (Fischer’s LSD test); *p < 0.05, **p < 0.01, ***p < 0.001. A representative of two experiments is shown.

We have previously reported Mit-A acts as an inhibitor of CRC by targeting the CSCs (23). We aimed to determine whether Mit-A along with αPD-L1 can cause increased cell apoptosis using orthotopic tumor biopsies grown as monolayer culture and compared with MC38 cell line. Firstly, the cytotoxic effect of Mit-A was evaluated with CellTiter-Glo assay and compared with the traditional colon cancer drug 5-FU (5-Fluorouracil) for MC38 monolayer cells. IC50 value for Mit-A and 5-FU was found to be 409.7 ± 8.06 nM and 2.55 ± 0.63 μM (**Supplementary Figure 1A**) respectively. For MC38 biopsies, the IC50 of Mit-A was found to be 1.27 ± 0.12 μM demonstrating the biopsies to be more resistant to Mit-A compared to immortalized cell line (**Figure 1A**). In our previous work, we have demonstrated that our in-house FiSS (Fiber-inspired smart scaffold system) forms tumoroids and expands CSCs (23, 28). We utilized these tumoroid cultures grown with orthotopic biopsies (**Supplementary Figure 1B**) recapitulating the *in vivo* microenvironment to test the efficacy of our combination treatment regimen (**Figure 1B**). CellTiter-Glo assay revealed a significantly higher decrease in cell viability for the combination compared to the Mit-A treatment in tumoroid cultures (**Figure 1B**).

In order to understand whether loss of cell viability is due to apoptosis, an Annexin V-binding assay was performed. We found that a monolayer of MC38 biopsies treated with Mit-A (300 and 600 nM) with or without αPD-L1 mAb (20 µg/ml) showed a significant increase in early apoptosis post 72 h of treatment in a dose-dependent manner (**Figures 1C, D**). Furthermore, the PD-L1 expression was measured post 72 h of 300 nM of Mit-A with or without αPD-L1 (20 µg/ml) and an increase of 7% was found with Mit-A treatment which was reversed in the presence of αPD-L1 treatment (**Figures 1E, F** **)**. These results suggest Mit-A sensitizes the tumor biopsy in part by increasing the PD-L1 expression, and causes enhanced early apoptosis upon combination treatment.

In order to understand whether the PD-L1 modulation is specific for MC38 cells or a general feature of epithelial cell lines we checked the PD-L1 expressions with/without treatment of Mit-A on two human other epithelial cell lines, HT29 (p53 mutant, K-RAS wild type, MSS) and HCT116 (p53 wild-type, K-RAS mutant, MSI). We observed a significant increase in the PD-L1 expression in HCT116 monolayer and tumoroid cultures upon treatment with Mit-A (10 and 25 nM) respectively (**Figure 1G**). Although not as pronounced as HCT116, a significant increase was observed in HT29 monolayer and scaffold when treated with Mit-A for 50 and 100 nM respectively (**Figure 1H**). The drug concentrations were chosen based on IC50 studies post 48 h of treatment (23). Thus, the modulation of PD-L1 is not restricted to MC38; Mit-A is shown to modulate in the human epithelial cell lines, HT29 and HCT116 that have been tested. Experiments with other epithelial cell lines are needed to find whether the PD-L1 modulation is a general feature of epithelial cells or not.

### Mit-A Promotes CD8^+^T Cell Activation *Ex Vivo* in the Presence of Orthotopic Immunosuppressive Biopsy

**Figure 2:**
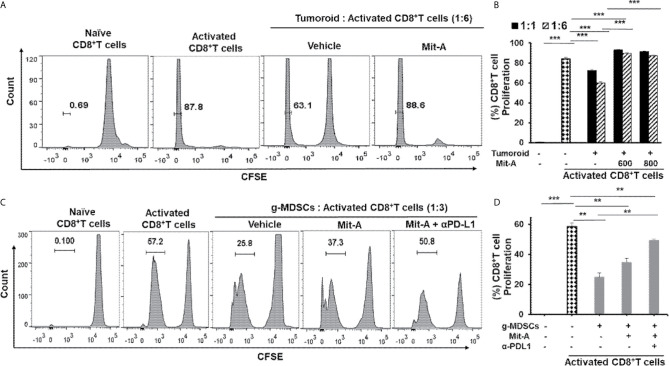
Mit-A enhances proliferation of activated CD8^+^ T cells and reverses the immunosuppression of tumoroid culture and g-MDSCs in combination with αPD-L1 mAb treatment. **(A, B)** CD8^+^ T cells isolated from naïve C57BL/6 mouse spleen were stained with CFSE proliferation dye, stimulated using CD3/CD28 activation beads and co-cultured with biopsy tumoroid (on Day 4 of tumoroid culture) in presence of IL-2 (30 U/ml). Mit-A was added to the co-culture immediately post T cell addition at various ratios to tumoroid cultures and T cell proliferation assessed by flow cytometry 72 h post addition. CFSE plots are shown in **(A)** and percent T cell proliferation plotted in **(B)**. **(C, D)** The sorted g-MDSCs isolated from the spleen of orthotopic MC38 tumor bearing mice were co-cultured with CFSE-labelled activated CD8^+^ T cells in 1:3 ratio (g-MDSC:T cells) in presence of Mit-A (600 nM) with or without αPD-L1 mAb (20 µg/ml). T cell proliferation was assessed by flow cytometry 72 h post treatment. CFSE plots are shown in **(C)** and percent T cell proliferation plotted in **(D)**. Data analyzed as mean ± SEM by Student’s t-test. **p < 0.01, ***p < 0.001. A representative of three experiments is shown.

Since the TME creates an immuno- suppressive effect on T cells, we aimed to determine whether Mit-A can reverse the immunosuppressive effects of the tumor milieu present in biopsy tumoroid-CD8^+^T cell co-culture. Orthotopic biopsy tumoroids were co-cultured with activated CD8^+^T cells (isolated from the spleen of naïve mice) in the presence of IL-2 at the 1:1 and 1:6 (tumoroid: CD8^+^T cell ratio) and the proliferation of the CFSE-stained CD8^+^T cells were measured post 72 h of Mit-A treatment. The proliferation of the activated T cells was hindered in presence of tumoroid reflecting the immunosuppressive effects of the biopsies grown on scaffolds, thus mimicking the TME. This inhibition of proliferation was reversed with Mit-A treatment and the effect was more pronounced with a 1:6 ratio compared to a 1:1 ratio (**Figures 2A, B** **)**. The differences in the CD8^+^ T cell proliferation from the vehicle to the Mit-A (600 nM) treated groups for 1:1 and 1:6 ratio were 20.4 and 29.7%, respectively and that for 800 nM Mit-A were 18.7 and 27.3%, respectively. Thus, though there is significant increase in both the ratios, the change was more prominent in the 1:6 ratio compared to the 1:1 for both drug concentrations.

### The Combination of Mit-A and αPD-L1 Suppresses the Immunosuppressive g-MDSCs and Reverses Their Suppressive Effect on T Cells

The TME becomes immunosuppressive through the activity of a diverse array of immunosuppressive immune cells, out of which MDSCs play a prominent role. Since MDSCs reprogram the tumor immunity by inhibiting T cell killing and other immunosurveillance (29), we reasoned that Mit-A would have an ameliorating effect in T cell activation. Since PD-L1 expression on MDSCs is known to have a suppressive effect on the immune response (17, 30), we tested whether the combination of αPD-L1 and Mit-A could reverse the mmunosuppressive effect leading to enhanced T cell proliferation. In this aspect, we performed a co-culture experiment where isolated CD11b^+^ cells from the spleen of an orthotopic tumor-bearing mouse were sorted for CD11b^+^Ly6G^+^Ly6C^low^ (granulocytic; g-MDSCs) and CD11b^+^Ly6G^-^Ly6C^+^ (monocytic; m-MDSCs) (**Supplementary Figure 1C**) and co-cultured with CD3/CD28 microbeads activated CD8^+^ T cells (from naïve mice spleen) in the ratio 1:3 (MDSC: T cell) and treated with Mit-A. Approximately 72 h post-treatment, T cell proliferation was found to be suppressed in the presence of the g-MDSCs which was reversed upon Mit-A treatment. The addition of αPD-L1 leads to a greater increase in T cell proliferation, thus revealing the effectiveness of the combination in tuning the T cell activation (**Figures 2C, D** **)**. Mit-A was not found to increase the T cell proliferation in the presence of m-MDSCs (**Supplementary Figure 1D**), and neither the addition of αPD-L1 showed an increase in T cell proliferation (data not shown), suggesting that g-MDSCs are the potential targets for the proposed therapy.

### The Combination of Mit-A and αPD-L1 Reduces the Tumor Burden in an Orthotopic Mouse Model and Arrests the Tumor Cell Proliferation

**Figure 3:**
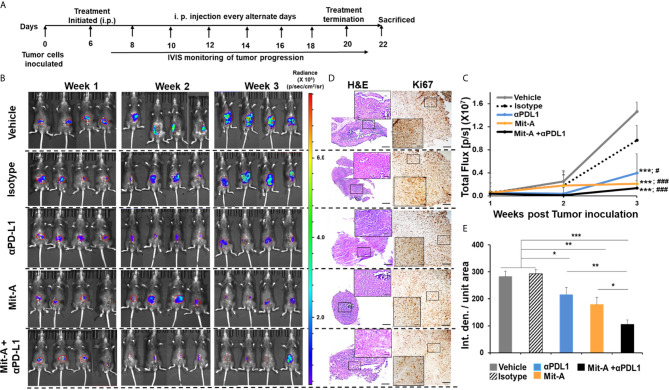
Combination of Mit-A and αPD-L1 reduces the tumor burden in orthotopic MC38 tumor-bearing mice. **(A)** Treatment scheme. Approximately 6 to 8 weeks old (female) wild-type C57BL/6 mice were anesthesized with isoflurane inhalation and the cecum was exposed *via* a lower abdominal incision. Approximately 2 × 10^5^ MC38-Luc cells suspended in 50 µl of PBS were injected subserosally using 30-G BD insulin syringe under the microscope (Day 0). On Day 6, the mice were divided into five groups and treatments were initiated (i.p.) every alternate day (n = 4/per group). The control group received the vehicle (PBS + DMSO), Mit-A group received 1.5 mg/kg/mice, isotype and anti-PD-L1 groups received 150 µg antibodies in PBS/mice and the combination groups got 1.5 mg/kg of the Mit-A + 150 µg of αPD-L1 mAb/mice. Treatment was continued until Day 21 after which the mice were sacrificed. **(B)** Tumor bioluminescence post 1, 2 and 3 week of MC38-Luc cell inoculation. Images captured by IVIS following i.p. injection of luciferin-D. IVIS images were quantified by Caliper Life Sciences Images software. **(C)** represents the total photon flux (p/s) measured by IVIS (* and # represent comparison with vehicle and isotype groups respectively for each group). **(D)** Representative images of the treated tumor sections showing H&E (left panel) and Ki67 (right panel), **(E)** Histograms showing Image J quantifications of Ki67 immunostaining. n = 3 mice/group. Scale bar 50 µm; inserts 10 µm. Data analyzed as mean ± SEM by Student’s t-test. *p < 0.05, **p < 0.01, ***p < 0.001. where IVIS, *In vivo* imaging system; Luc, luciferase; p/s, photon/second; PBS, phosphate buffered saline; DMSO, dimethyl sulfoxide; Int. den., integrated density. The data is representative of three experiments. “###” denotes the significance between isotype and the other groups.

The therapeutic effect of Mit-A alone or in combination with αPD-L1 mAb was evaluated *in vivo*. To determine whether the combination of Mit-A with αPD-L1 mAb inhibits tumor growth and reduces the tumor burden, a luciferase reporter expressing MC38 (MC38-Luc) was injected subserosally in the cecum of the C57BL/6 mice in 50 µl of PBS. Treatment was initiated on Day 6 intraperitoneally and continued every alternate day where control group received vehicle (DMSO/PBS). Isotype and αPD-L1 groups received 150 ug/mouse of IgG and αPD-L1, respectively in PBS. Approximately 1 mg/kg of Mit-A was administered with or without αPD-L1 mAb in PBS (**Figure 3A**) (n = 4 mice per group). Tumor progression was monitored every week by bioluminescence using IVIS (**Figure 3B**). On Day 22, mice were sacrificed, tumors collected, weighed, and processed. No significant changes in the body weight were observed during the span of the experiment except for an increase in the weight of the control and IgG treated groups due to tumor growth. We found a significant decrease in tumor growth with Mit-A treatment. Combining it with αPD-L1 mAb resulted in a better tumor growth inhibition than the control and isotype. A reduction in the total flux of the IVIS images of the treatment groups demonstrated that the combination treatment led to higher tumor growth arrest compared to the rest of the groups (**Figure 3C**). We found significant decreases in the tumor growth in both the αPD-L1 mAb monotherapy and combination treatment groups compared to the isotype and vehicle controls. Although the combination group exhibited the most significant decrease in tumor growth, this difference between combination and αPD-L1 mAb treatment groups was not significant. The results aligned with the tumor growth images captured post sacrifice (data not shown). Histological (H & E) and immunohistochemical (Ki67) staining were performed in tumor sections following different treatments (**Figure 3D**). Tumors treated with the combination therapy were found to have the smallest tumors with significantly reduced Ki67 expression compared to the monotherapy treated groups and control (**Figure 3E**). We also tested our combination of Mit-A/αPD-L1 in CT26-bearing (murine colon cancer cell line (p53 wild-type, K-RAS mutant, MSS) subcutaneous tumor model in Balb/c mice. CT26 tumor model showed significantly decreased tumor growth compared to the control (vehicle) group and monotherapy groups (**Supplementary Figures 5A, B** **)**.

### PD-L1 Blockade in Combination With Mit-A Increases T Cell infiltration and Decreases Immunosuppressive Tregs

**Figure 4:**
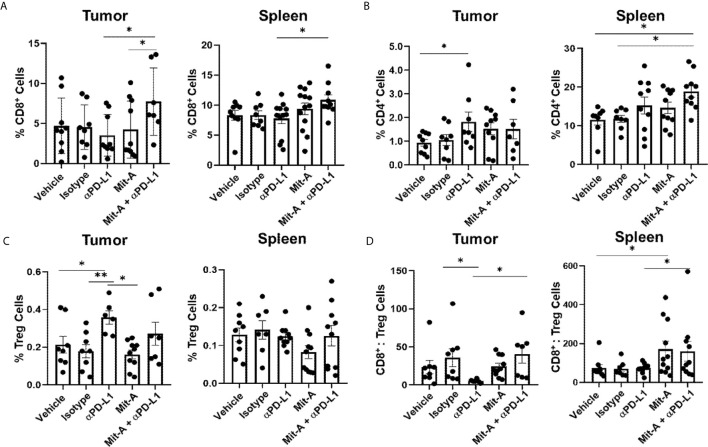
PD-L1 blockade in combination with Mit-A increases T cell infiltration and decreases immunosuppressive Tregs. Flow cytometry analysis of tumor and spleen collected from the orthotopic tumor-bearing mice treated with vehicle, Isotype (IgG), Mit-A w/o αPD-L1 mAb. All cell populations were gated out of CD45^+^ live cells. **(A)** Percentage of CD8^+^ T cells (CD3^+^CD8^+^) for tumor (left) and spleen (right); **(B)** Percentages of CD4^+^ T cells (CD3^+^CD4^+^) for tumor (left) and spleen (right); **(C)** Tregs representing percentage of CD3^+^CD4^+^CD25^+^FoxP3^+^ for tumor (left) and spleen (right) were analyzed and compared among the treatment groups. **(D)** CD8^+^ T cells:Tregs ratio for tumor (left) and spleen (right), respectively shown. Each dot represents one individual mouse. Data pooled of two independent experiments (minimum n = 4 per group for each experiment) and analyzed as mean ± SEM by One-way ANOVA (Fischer’s LSD test). *p < 0.05, **p < 0.01.

Both spleen and tumors from the five groups (vehicle control, IgG, αPD-L1, Mit-A, and Mit-A + αPD-L1) were processed, collected as single-cell suspension, and assessed by flow cytometry analysis. Out of live cells, CD45^+^ cells were gated for CD3^+^CD8^+^ and CD3^+^CD4^+^ T cells and Tregs were analyzed from CD4^+^CD25^+^FoxP3^+^ cells (**Supplementary Figure 2A**). A significant increase in CD8^+^ T cell population for the combination-treated group was found compared to αPD-L1 treatment alone both in spleen and tumor (**Figure 4A**). Although an increase in CD4^+^ T cell population was found in the spleen, no similar increase was found in the tumor (**Figure 4B**). When analyzed in tumor, although a significant decrease in Tregs was found for Mit-A compared to αPD-L1 treatment, the combination treatment showed no significant decrease (**Figure 4C**). However, a higher CD8:Tregs ratio was found for the Mit-A + αPD-L1 treatment group compared to the αPD-L1 monotherapy in both tumor and spleen (**Figure 4D**) suggesting that the combination treatment leads to higher T cell infiltration with suppression of the immunosuppressive Tregs cell population.

### Mit-A in Combination With αPD-L1 Leads to a Decrease in the g-MDSCs and M2 Macrophages *In Vivo*

**Figure 5:**
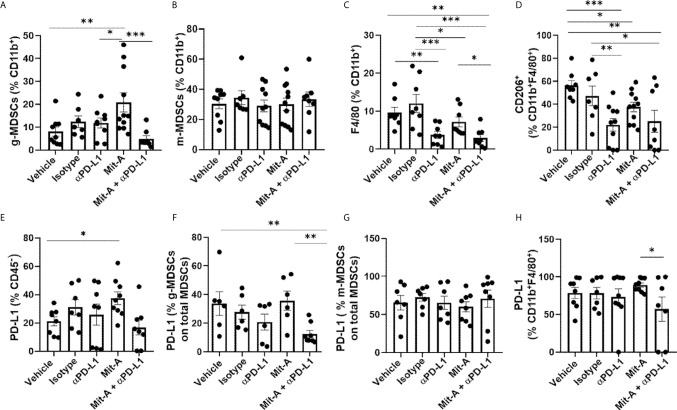
Mit-A and anti-PD-L1 combination treatment decreases the immunosuppressive g-MDSCs population. Mit-A sensitizes orthotopic tumor and immune cells by modulating PD-L1 expression *in vivo*. Flow cytometry analyses of tumor from the MC38 orthotopic tumor bearing mice from the five groups treated with vehicle, IgG (isotype), αPD-L1 mAb, Mit-A and Mit-A + αPD-L1 mAb where all cell populations were gated out of CD45^+^ live cells. **(A)** Percentage of g-MDSCs (CD11b^+^Ly6G^+^Ly6C^low^ (granulocytic)) and **(B)** m-MDSCs ((CD11b^+^LyG^-^Ly6C^+^ (monocytic) populations from tumor of MC38 orthotopic-tumor bearing mice shown. **(C, D)** Percentage of CD11b^+^F4/80^+^ macrophage and CD206^+^CD11b^+^F4/80^+^ anti-inflammatory M2-macrophage populations, respectively from tumor treated with the five treatment groups shown. **(E–H)** Percentages of PD-L1 expression on CD45^-^ tumor cells **(E)**, g-MDSCs **(F)**, m-MDSCs **(G)** from tumor (calculated out of total MDSCs) and tumor CD11b^+^F4/80^+^ macrophage population **(H)**. Each dot represents one mouse in every group. Data pooled of two experiments (minimum of n = 3 per group for each experiment) and analyzed as mean ± SEM by One way ANOVA (Fischer’s LSD test). *p < 0.05, **p < 0.01, ***p < 0.001.

MDSCs were analyzed in both tumor and spleen from CD45^+^CD11b^+^ populations gated from CD45^+^ live cells (**Supplementary Figure 2B**), and the granulocytic (CD11b^+^Ly6G^+^Ly6C^low^) and monocytic (CD11b^+^Ly6G^−^Ly6C^+^) subpopulations were analyzed for the five treatment groups (**Figures 5A, B** **)**. A significant decrease in the g-MDSC population was found for the combination group compared to the Mit-A alone group. Although a marginally significant increase was observed with the Mit-A single treatment compared to the control and αPD-L1 treatment groups, an overall decrease in the g-MDSC subpopulation was observed when αPD-L1 was combined with Mit-A (**Figure 5A**). No observable differences in the m-MDSC subpopulation were found in the tumor reflecting that the treatment did not impact m-MDSC subtypes in this model (**Figure 5B**). For the spleen, we observed a decrease in the g-MDSC cells in the combination and αPD-L1 treated groups compared to the IgG treated group (**Supplementary Figure 3A**). No significant difference between the Mit-A monotherapy and combination group was found in the spleen, thus emphasizing the combination treatment’s effect on the g-MDSC population in the TME (decreasing its fraction and thus its immunosuppressive effects) (**Figure 5A**). However, a significant decrease in the splenic m-MDSCs was, observed with the combination and αPD-L1 treatment groups compared to the IgG group (**Supplementary Figure 3B**).

TAMs are also associated with immunosuppression, and their population is found to be increased in tumor growth and their release of anti-inflammatory cytokines. CD11b^+^F4/80^+^ cells representing the total macrophage population were significantly reduced with the combination treatment as compared to the single drug Mit-A, IgG-treated, and vehicle (control) groups (**Figure 5C**). However, no statistically significant difference was found between the αPD-L1 treated group and the combination-treated group predicting that the association of Mit-A with αPD-L1 was not able to suppress the M2 macrophage population (CD11b^+^CDF4/80^+^CD206^+^) as compared to monotherapy. Although, the M2 population was suppressed in all treatments compared with the control and IgG groups (**Figure 5D**). Thus, although the combination suppressed the g-MDSCs and overall macrophage population, a robust decrease in the M2 subpopulation was not evident compared to the monotherapy.

### Mit-A Sensitizes the Tumor and Immune Cells by Modulating the PD-L1 Expression

In our *ex vivo* studies, we observed an increased PD-L1 expression of the tumor cells upon treatment with Mit-A alone. A similar observation *in vivo* of PD-L1 expression on CD45- tumor cells revealed that Mit-A sensitizes the tumor cells by increasing its expression, which was decreased when, combined with αPD-L1 therapy (**Figure 5E**). Since we found the combination had a suppressive effect on the g-MDSCs population, the PD-L1 expression on tumor (**Figure 5F**) and spleen (**Supplementary Figure 3C**) g-MDCS was compared among the treatment groups. Percentage of PD-L1 expression on the g-MDSC when calculated among the total MDSCs (CD11b^+^Ly6G^+^Ly6C^low^ + CD11b^+^Ly6G^-^Ly6C^+^) in the tumor was found to be decreased with the combination treatment compared to the Mit-A alone. We did not observe any change in the PD-L1 expression for a similar population on the spleen (**Supplementary Figure 3C**). This data suggests that the combination therapy specifically targeted the tumor g-MDSCs, which are known to have suppressive effects. No change in the PD-L1 expression for tumor m-MDSCs was found (**Figure 5G**). However, although the PD-L1 expression on the tumor CD11b^+^F4/80^+^ macrophages was found to be decreased in the combination treatment compared to the Mit-A alone, no significant changes were observed when compared to the αPD-L1 mAb monotherapy (**Figure 5H**).

**Figure 6:**
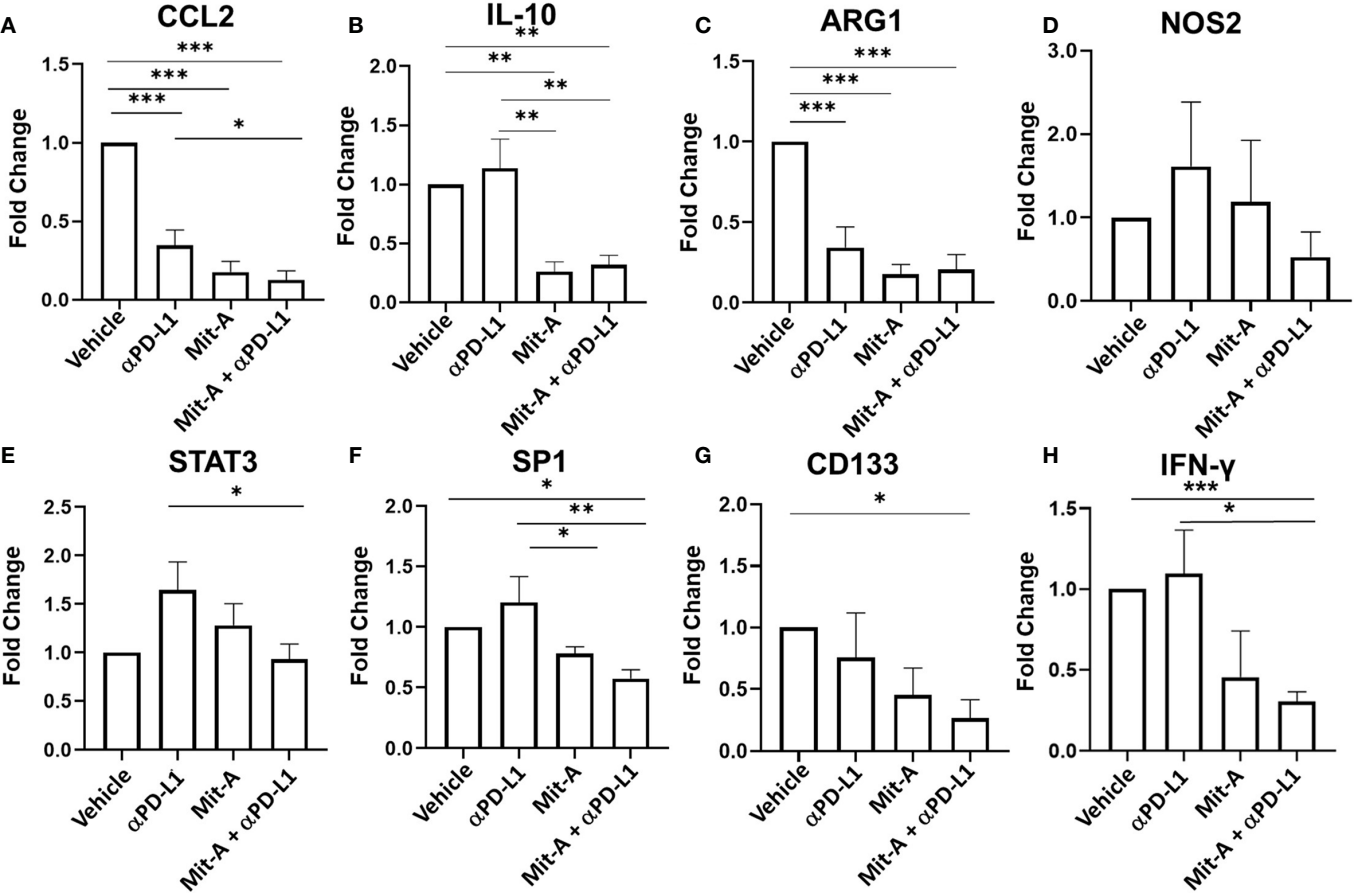
Mit-A in combination with αPD-L1 mAb suppresses the g-MDSCs along with CSCs. Total RNA was isolated from the MC38 orthotopic tumors of the treatment groups (vehicle, isotype (IgG), Mit-A with or without αPD-L1 mAb), and subjected to qPCR analyses of **(A)** *CCL2*, **(B)** *IL-10*, **(C)** *ARG1*, **(D)** *NOS2*, **(E)** *STAT3*, **(F)** *SP1*, **(G)** *CD133* and **(H)** IFN-γ transcripts (n = 3). The vehicle and group was normalized and other groups were compared with the normalized control (vehicle) group. Gene expressions of vehicle and isotype were found to be similar. Data analyzed as mean ± SEM by One way ANOVA (Fischer’s LSD test). *p < 0.05, **p < 0.01, ***p < 0.001. A representative of two experiments is shown.

Since MDSCs migrate to the tumor site by C–C motif ligand 2 (CCL2) driven pathway and are involved in M2 macrophage polarization we analyzed CCL2 and IL-10 (which are anti-inflammatory cytokines released by M2 and also by MDSCs) (31) gene expression in the tumor sections collected from the treatment groups. A decrease in CCL2 and IL-10 expression was observed in the combination group compared to control and αPD-L1 treatment groups (**Figures 6A, B** **)**. Since arginase-1 (ARG-1) and nitric oxide synthase 2 (NOS2) activation leads to the suppressive effects of MDSCs (26, 29), we next checked the expression of ARG1 and NOS2 in the tumor collected from the treatment groups. We found a significant decrease in the transcript level of *Arg1* compared to the control in the monotherapy and the combination group. We did not find any significant differences, however, in the *Nos2* transcript level (**Figures 6C, D** **)**. This data suggested that Mit-A with/without αPD-L1 has a direct effect on the infiltrating g-MDSCs at least partially in terms of reversal of T cell proliferation. Furthermore, activation of STAT3 is associated with MDSC activation in CRC and its phosphorylation is correlated with tumor growth (29). A significant decrease in the STAT3 expression in the tumor with the combination treatment was found compared to the αPD-L1 therapy validating that MDSCs are suppressed by Mit-A in addition to αPD-L1 (**Figure 6E**). We have previously reported CSC as potential targets of Mit-A (23). In an attempt to test whether the combination therapy apart from targeting MDSCs and macrophages can foster the CSC killing, CD133 and SP1 gene expression was measured in the tumor sections revealing a significant decrease in both (**Figures 6F, G** **)**. Thus, Mit-A which not only targets CSCs and inhibits SP1, continues to show these activities when used in combination with αPD-L1. Since IFN-γ produced by T cells is known to upregulate PD-L1 expression (32), we checked the IFN-γ expression in the tumor lysates of the treatment groups. We found a decrease of IFN-γ in the combination therapy group compared to the PD-L1 monotherapy treated group (**Figure 6H**). 

## Discussion

In the majority of CRC patients, CB therapy has not been successful due to the presence of microsatellite stability and lower mismatch repair deficiencies (5). Although it has been proven to regress tumor growth in MSI-H patients, which comprise 5–10% of the patient population, most do not respond to the single therapy (7). As a result, the combination approach for superior clinical response holds potential for overall survival and progression-free survival (4, 33). While pre-clinical studies for various combination therapies are being tested in subcutaneous models, the lack of tissue-specific TME with its underlining heterogeneous immune profiles in these models makes them unsuitable for an accurate evaluation of these therapies (34). In this context, orthotopic tumors contain a native local tumor milieu enhancing the clinical relevance of this model for testing immunomodulatory agents and checkpoint blockers as combination approaches (35). While the MC38 orthotopic tumor model serves as a syngeneic murine model generated from wt-KRAS, MSI-H, and p-53 mutant MC38 cell line, we demonstrated as a proof-of-concept, the combination treatment could be effective in studying the mechanisms of immune modulation in response to CB (36). The effects of the combination treatment is not limited to MC38 orthotopic tumors. We also tested our Mit-A and αPD-L1 combination in CT26- bearing subcutaneous tumor model with MSS genetic mutations in Balb/c mice and found decrease in tumor growth in the combination groups compared to the control and monotherapy groups.

MC38 cells are known to express PD-L1 which gets up-regulated in the presence if IFN-γ *in vitro* (37, 38), and in our study, we found increased expression of PD-L1 on MC38 orthotopic tumor biopsies in response to the immunomodulatory drug, Mit-A. Chemoresistance due to significant upregulation of PD-L1 expressions in cancer cells by various drugs, such as doxorubicin (DOX), Oxaliplatin (OXA, a DNA alkylating agent), Paclitaxel (PACLI, a tubulin inhibitor), Irinotecan (IRI, a topoisomerase 1 inhibitor) has been found and the role of ERK activation increase has been correlated to the overexpression of PD-L1 (14, 39). These effects have been attributed to the sensitizing ability of cancer cells to αPD-L1 therapy, thus altering the “cold” TME to “hot” one (40, 41). The expression of PD-L1 on tumor and immune cells correlates to CB therapy’s objective response and clinical outcome. Thus, the level of PD-L1 expressions and its regulation has become a predictive marker for personalized mono- or combination CB treatment (11). In cancer patients, where the PD-L1 level is low, the CB therapy fails. We found that Mit-A treatment increases in PD-L1 expression in cancer and immune cells, and sensitizes the tumor to anti-PD-L1 therapy. However, the mechanism of action is not clear.

Amongst several intrinsic and extrinsic factors that regulate PD-L1 expression in various cancers, DNA methylation of the PD-L1 promoter has been suggested recently in cancer malignancies (42). For instance, TGFβ1 has been shown to induce decrease expression of DNA-methyltransferase 1 (DNMT1) and PD-L1 promoter demethylation, leading to PD-L1 overexpression in lung cancer cells that were undergoing EMT (43). Thus, hypomethylating agents have a direct effect on the PD-L1 expression and thus the epigenetic hypomethylating agents are potential candidates for increasing the combination CB therapy (42).

It has been found that Mit-A reduces the CpG island methylation and inhibits 5’-cytosine-DNA-methyltransferase which is related to anti-metastatic tumor-suppressor genes in lung cancer cells (44). Additionally, Mit-A blocks SP1 from binding to DNA and acetylated SP1 is known to inhibit PTEN expression through binding to the PTEN core promoter (45). PTEN loss activates PI3K signaling that leads to an increase in PD-L1 expression (42). However, the precise mechanism of how Mit-A increases PD-L1 in MC38 tumor cells still remains to be elucidated. Thus, Mit-A which increases the PD-L1 expressions in various epithelial CRC cells, stands as an augmenting agent for CB therapy. Herein, we demonstrated that when orthotopic tumor-bearing mice were treated with Mit-A in addition to checkpoint-blocker αPD-L1, CD8^+^ T cell infiltration in the tumor increased thereby arresting its growth. The resistance to αPD-L1 monotherapy thus could be overcome by combining Mit-A in the treatment regime.

Immature myeloid cells tend to differentiate into macrophages, granulocytes, and dendritic cells under normal physiological conditions (18). However, in cancer and other pathologic conditions, their differentiation is hampered resulting in the development and recruitment of MDSCs which are activated by suppressing the T cell infiltration locally thus aiding the tumor cells to evade immunosurveillance (46). The two subtypes of MDSCs, g-MDSCs and m-MDSCs hinder the effector T cell function by multiple pathways. Apart from MDSCs, the tumor stroma is infiltrated with TAMs, which also assist in abrogating the anti-tumor immunity (13). The co-inhibitory PD-1 receptors on the T cells with the association with its ligand PD-L1 helps in apoptosis, anergy, and exhaustion of T cells, thereby promoting tumor growth and metastasis (8). Not only on tumor cells, but PD-L1 expressions on immune cells (myeloid) have been reported to be responsible for CD8^+^T cell suppression in the murine CRC model (37, 47). Therefore, blocking PD-L1 on these cells along with tumor cells with checkpoint inhibitors holds potential for anti-tumor immunity (37). Our study demonstrated the reversal of the inhibition of immunosuppression of T cell proliferation by g-MDSC *via* Mit-A and αPD-L1 combination in monolayer co-culture platform *in vitro* as well as in the increase of CD8:Treg ratio *in vivo*. We showed that our tumoroid platform when co-cultured with T cells mimics an immunosuppressive environment for drug testing. As it has been found MSI-H patients who respond to CB therapy are infiltrated with increased CD8^+^ T cells along with other factors such as elevated neoantigens and genetic mutations (48), increase in CD8^+^ T cells as found in our model would aid in improved response to CB. Our data suggest that patients with MSS genetic mutations could respond to this combination therapy which likely to alter the TME *via* MDSC and TAMs inhibition.

As a monotherapy, Mit-A was unable to block PD-L1 expression within the TME. A dose-dependent increase in PD-L1 expression was observed with Mit-A treatment *in vitro* for tumor biopsies (data not shown) which reflected in the increased PD-L1 expression in the CD45^-^ tumor cells from tumor-bearing mice treated with Mit-A, thus demonstrating the capacity of Mit-A to sensitize orthotopic tumor cells for improved checkpoint blocking therapy. Although the PD-L1 expression was found to be increased in tumor cells with Mit-A monotherapy, the effect was reversed in presence of the combination suggesting that the cytotoxicity of Mit-A on tumor cells is immunomodulating the TME for enhanced infiltration of T cells *via* application with the αPD-L1 mAb. The plausible mechanism could be through immunogenic cell death since Mit-A is known to increase tumor sensitivity due to DNA damage (49); this could affect in enhanced combination therapy with CB (50).

We checked the PD-1 on CD8^+^, CD4^+^ and Tregs cells (**Supplementary Figures 6A–C**). PD-1, which is a T cell inhibitory as well as activation marker, was found to be increased on CD8^+^ T cells within the combination treated tumors compared to Mit-A treated group (**Supplementary Figure 6A**). However, Mit-A treatment alone led to a decrease in CD8^+^ PD-1^+^ cells compared to the control tumors. These data suggest that although the PD-L1 expression was decreased in tumor for combination treatment, PD-1 increased on CD8^+^ T cells when Mit-A was combined with αPD-L1. This result is consistent with the activation of cytotoxic CD8^+^ T cells. However, we currently do not know if these T cells remain activated persistently or proceed to an exhausted state. Further studies will be required to answer this question. No significant changes in the PD-1^+^ CD4^+^ T cells were observed amongst the treatment groups (**Supplementary Figure 6B**) suggesting a lack of helper T cell participation with these treatments. A significant decrease of PD-1^+^ on Tregs (**Supplementary Figure 6C**) was observed for Mit-A treated group compared to the control group correlating with lesser activation of these immunosuppressive cell populations.

Intratumoral CCL2 expression levels have been reported in CRC patients and accumulation of MDSC induced by CCL2 correlated with the development and growth of colon adenoma (51). Mainly polymorphonuclear MDSCs which represent the granulocytic population are regulated by CCL2 in a STAT-3 dependent manner causing T cell suppression (31). Mit-A alone and in combination led to a decrease in the CCL2 gene expression *in vivo* as compared to the control tumors suggesting the g-MDSCs are the key targets for the therapy.

TAMS with M2 phenotype is responsible for angiogenesis, tumor promotion and adaptive immunity suppression (27). These M2 macrophages known for drug resistance in CSCs, act *via* STAT3 activation (52). Consistently, reduced expression of CD133, a marker for CSCs was observed both with Mit-A w/o αPD-L1 *in vivo*. As evidenced by the decrease in the M2 macrophages (CD206^+^ F4/80^+^) by Mit-A along with αPD-L1 treatment, our combination therapy was able to block the resistant CSCs *via* M2 suppression. Furthermore, tumor lysate IFN-γ measurement by RT-PCR (**Figure 6H**) was found to be decreased in the combination group compared to the controls. We argued this observation correlated with the decrease in the PD-L1 expressions in the tumor by combination treatment.

Herein, we have investigated the effect of Mit-A on the major tumor-infiltrating immune cells (T-cells, MDSCs, macrophages). Other populations (NK cells, DCs) have very low abundance in the MC38 tumor (53). Since we observed a decrease in the g-MDSCs with the combination treatment, the PD-L1 percentages in those subset populations were checked in particular. No significant change in the NK or dendritic cell population were observed among the treatment group. Granulocytic MDSCS and macrophages contribute to the immunosuppressive populations affecting the TME as observed in most cancer patients (29). Thus, we rationalized that these populations would be affected most compared to other cell populations and our findings largely corroborated this hypothesis.

## Conclusion

Herein we have demonstrated the efficacy of Mit-A in overcoming the resistance of αPD-L1 monotherapy by sensitizing the tumor cells when treated in combination by targeting the immunosuppressive TME in the MC38-orthotopic mouse model. Our findings suggest that suppression of g-MDSCs by blocking their PD-L1 receptors and thus increasing the T cell infiltration with the combination strategy could be a potential therapeutic modality for MSS CRC patient cohorts.

## Data Availability Statement

The original contributions presented in the study are included in the article/**Supplementary Material**. Further inquiries can be directed to the corresponding author.

## Ethics Statement

The animal study was reviewed and approved by Institutional Animal Care and Use Committee (IACUC) of the University of South Florida.

## Author Contributions

SM and SSM conceptualized the study and corrected the manuscript. RD executed *in vitro* and *in vivo* experiments, sample preparation, Flow cytometry and PCR studies, data analyses and writing the manuscript. RK, RG, and MH helped in flow cytometry studies. KM helped in immunohistochemistry and SB provided thoughtful suggestions. All authors contributed to the article and approved the submitted version.

## Funding

This work was supported by Veterans Affairs Merit Review grant (BX003413) to SM, Research Career Scientist Awards to SM (IK6BX004212) and SSM (IK6BX003778), and Circle of Hope Research Initiative Grant Award to RD.

## Author Disclaimer

Though this report is based upon work supported, in part, by the Department of Veterans Affairs, Veterans Health Administration, Office of Research and Development, the contents of this report do not represent the views of the Department of Veterans Affairs or the United States Government. This work has been supported in part by the Fred Wright Jr. Flow Cytometry Core at University of South Florida College of Medicine.

## Conflict of Interest

SM (founder and scientific advisor) and SSM (founder and scientific advisor) have an equity interest in Transgenex Nanobiotech Inc. The terms of this arrangement have been reviewed and approved by the USF in accordance with its conflict of interest policies.

The remaining authors declare that the research was conducted in the absence of any commercial or financial relationships that could be construed as a potential conflict of interest.

## Publisher’s Note

All claims expressed in this article are solely those of the authors and do not necessarily represent those of their affiliated organizations, or those of the publisher, the editors and the reviewers. Any product that may be evaluated in this article, or claim that may be made by its manufacturer, is not guaranteed or endorsed by the publisher.
