# Supplementary figures and images for: Combination Therapy of Mithramycin A and Immune Checkpoint Inhibitor for the Treatment of Colorectal Cancer in an Orthotopic Murine Model
Source: Front Immunol. 2021 Jul 26;12:706133. doi: 10.3389/fimmu.2021.706133 (PMC8350740; doi:10.3389/fimmu.2021.706133)

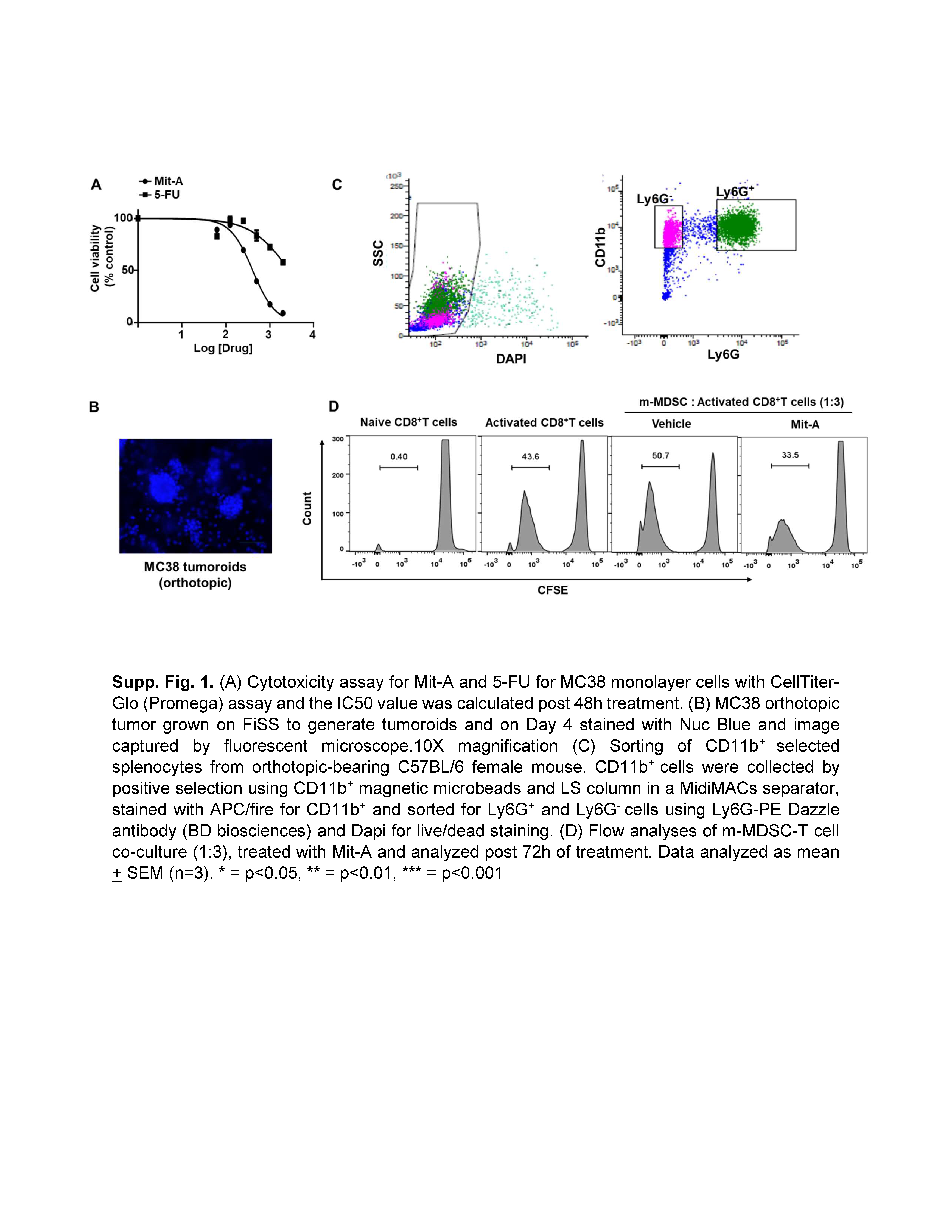

Supplement: Supplementary file 1 [file Image_1.tif]

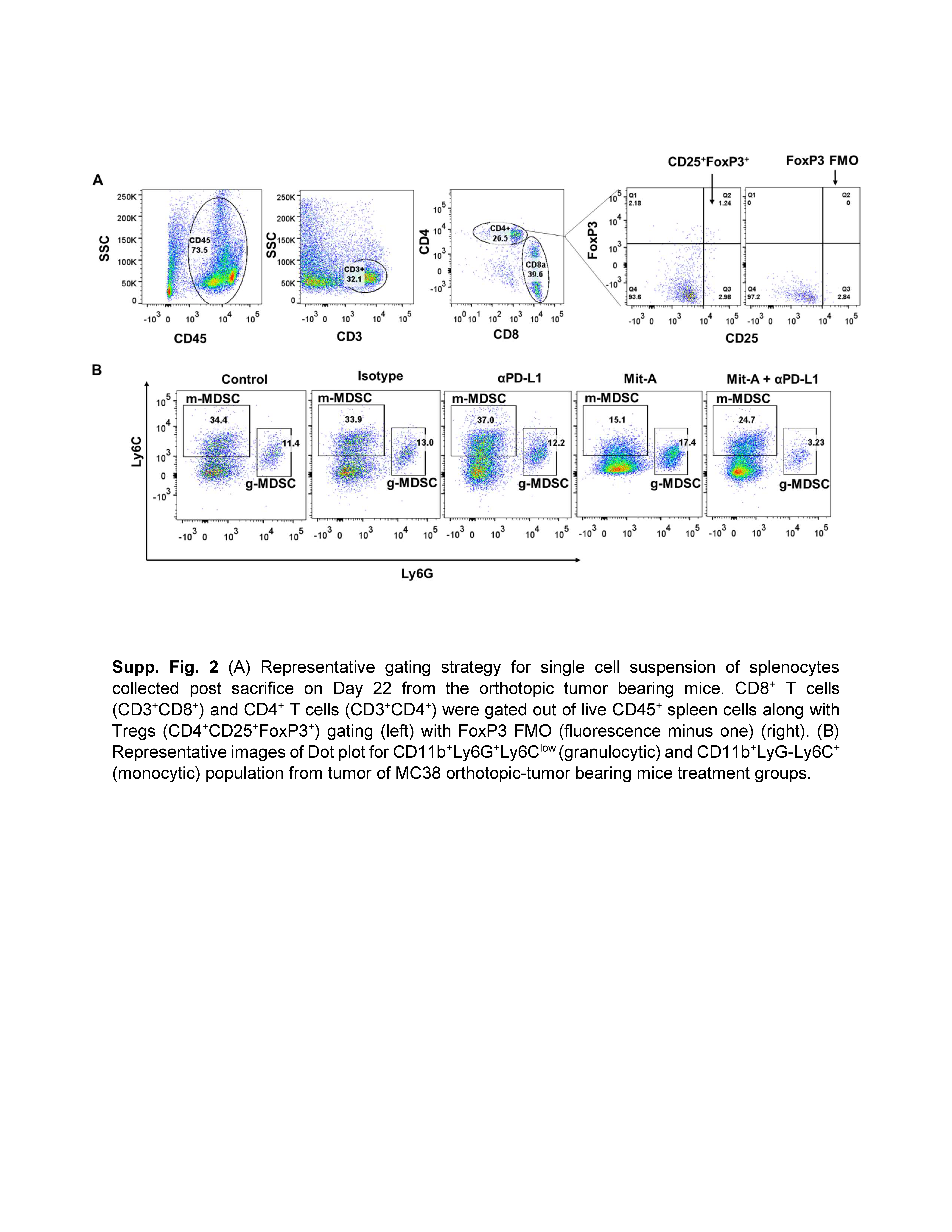

Supplement: Supplementary file 2 [file Image_2.tif]

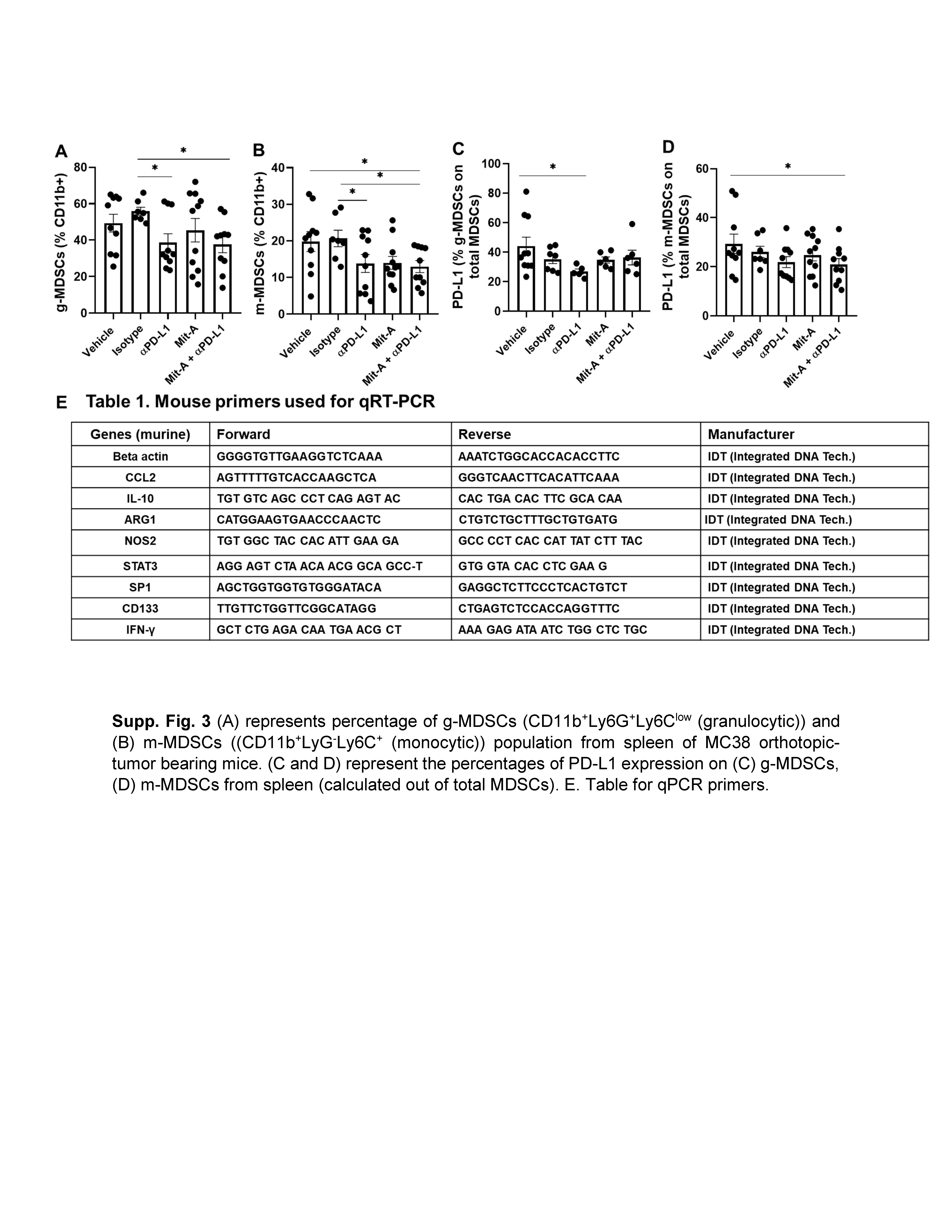

Supplement: Supplementary file 3 [file Image_3.tif]

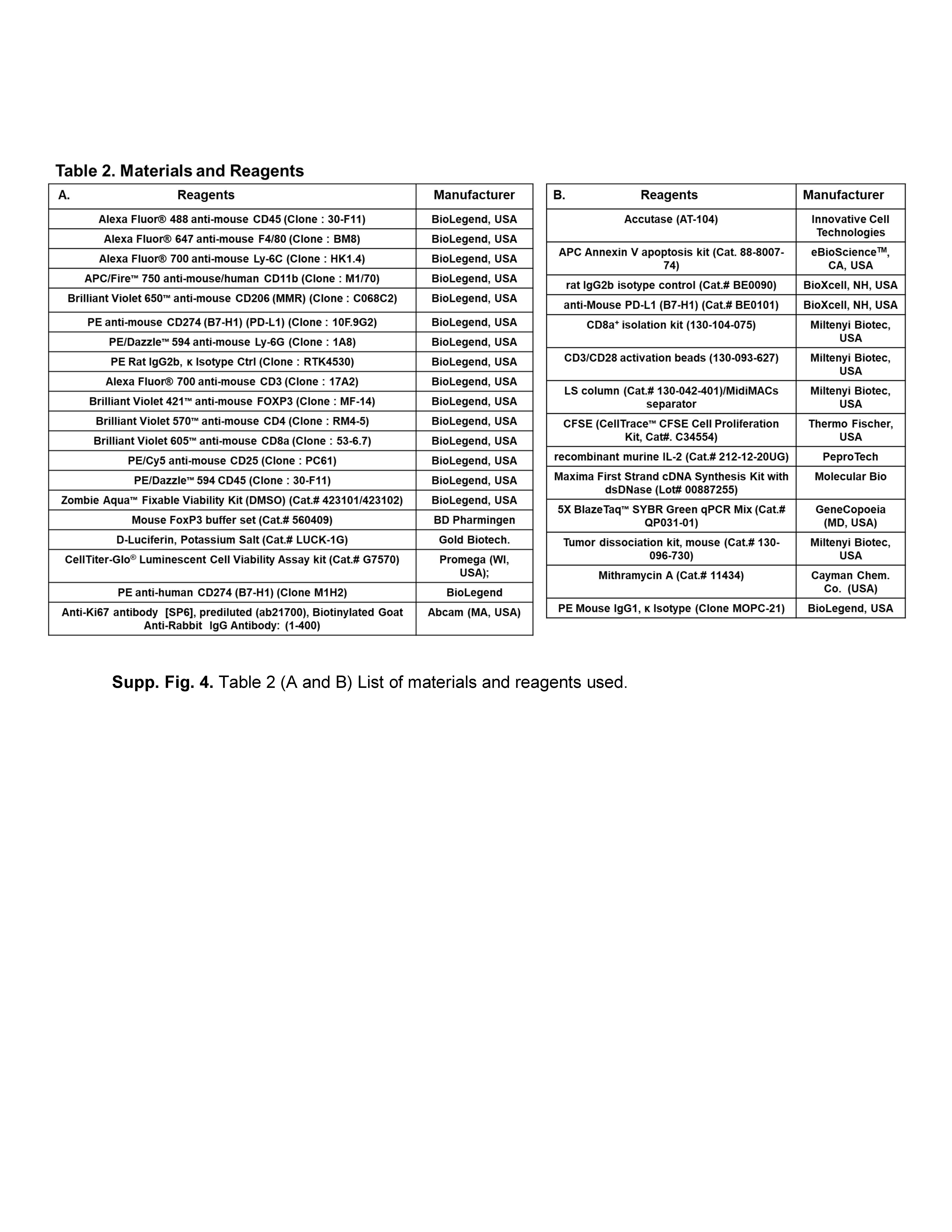

Supplement: Supplementary file 4 [file Image_4.tif]

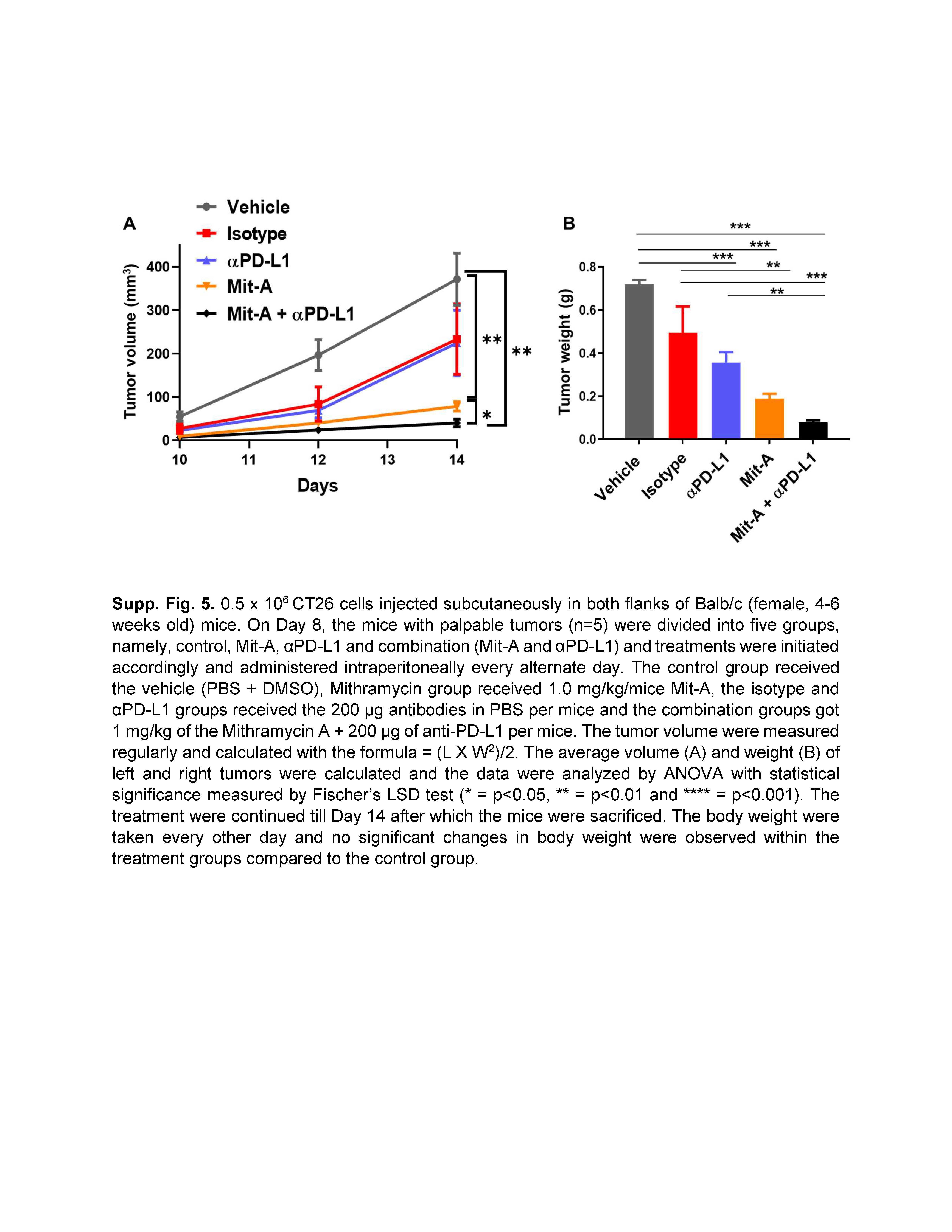

Supplement: Supplementary file 5 [file Image_5.tif]

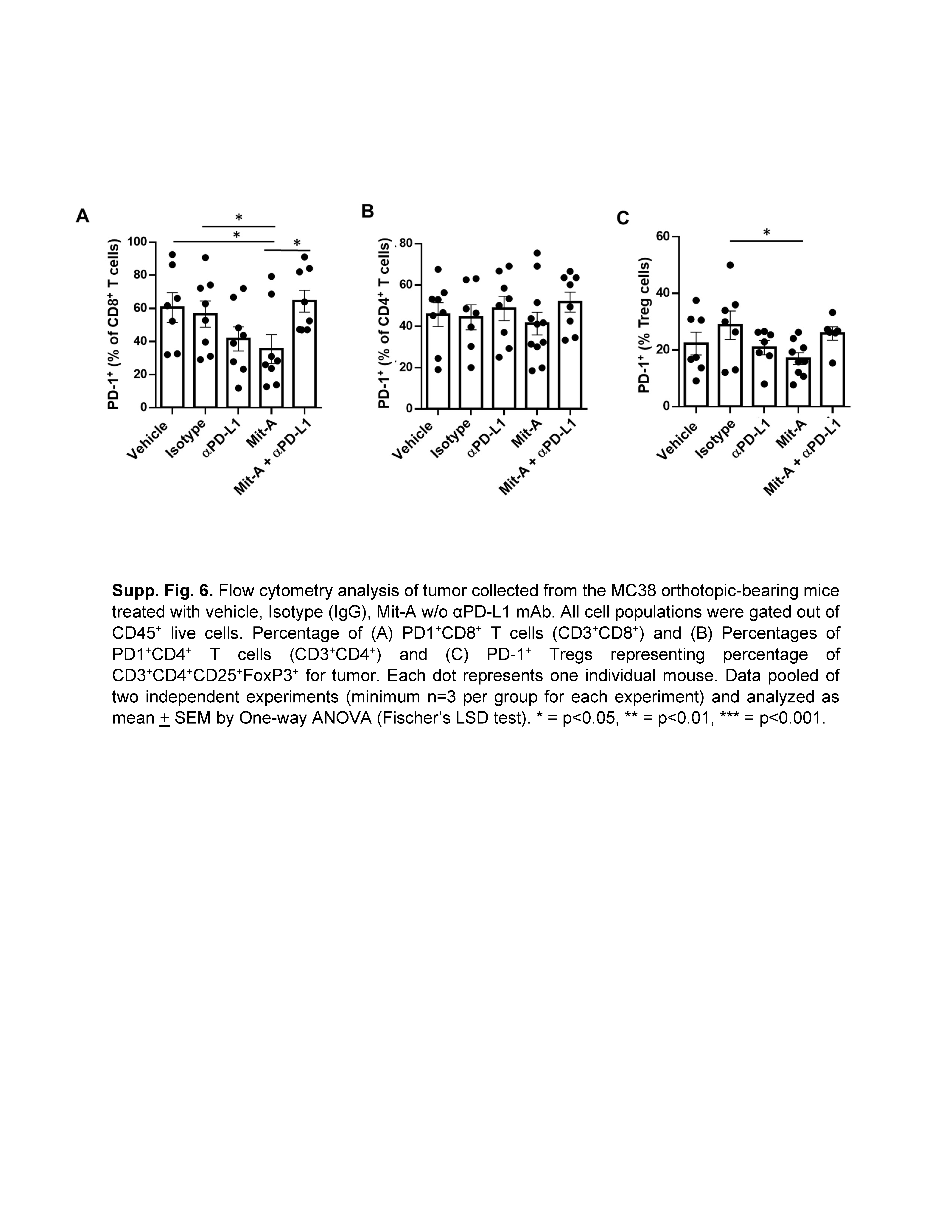

Supplement: Supplementary file 6 [file Image_6.tif]
